# Menthol mouth rinsing and performance in elite football referees in the heat: A study protocol for a randomized crossover trial

**DOI:** 10.1016/j.conctc.2023.101202

**Published:** 2023-08-22

**Authors:** Maria Roriz, João Brito, Filipe J. Teixeira, Konstantinos Spyrou, Vitor Hugo Teixeira

**Affiliations:** a Faculty of Nutrition and Food Sciences, University of Porto (FCNAUP), 4150-180, Porto, Portugal; b Portugal Football School, Portuguese Football Federation, Oeiras, Portugal; c Interdisciplinary Center for the Study of Human Performance (CIPER), Faculdade de Motricidade Humana, Universidade de Lisboa, Estrada da Costa, 1499-688, Cruz-Quebrada, Portugal; d Atlântica, Instituto Universitário, Fábrica da Pólvora de Barcarena, 2730-036, Barcarena, Portugal; e UCAM Research Center for High Performance Sport, UCAM Universidad Católica de Murcia, Murcia, Spain; f Facultad de Deporte, UCAM Universidad Católica de Murcia, Murcia, Spain; g Research Centre in Physical Activity, Health and Leisure (CIAFEL), Faculty of Sports, University of Porto (FADEUP), 4200-450, Porto, Portugal; h Laboratory for Integrative and Translational Research in Population Health (ITR), 4050-600, Porto, Portugal

**Keywords:** Football referees, Heat, Internal cooling, Menthol, Sports performance

## Abstract

**Background:**

Within professional European competitions, matches can be played in extreme environmental temperatures, ranging from −5 °C to +30 °C in different countries. Furthermore, the World Cups are usually played in the summer months, when temperatures can exceed 35 °C, increasing physiological stress. Practical and cost-effective cooling strategies may be implemented to help players and referees to cope with exercising in the heat. No study has evaluated the effect of non-thermal internal cooling techniques regarding performance responses on elite football referees, so far. This study aims to analyse the effects of a menthol mouth rinse regarding physical, physiological, and perceptual performance in elite male football referees, during a 90-min football protocol in the heat.

**Methods:**

At least thirteen male football referees will be recruited to perform two intermittent football protocols, separated by no less than 7 days. After passing the eligibility criteria, the participants will be randomly assigned to 1 of 2 beverages: (1) intervention - menthol solution 0.01% and (2) placebo - noncaloric berry-flavored solution, both at room temperature. The beverages will be given before warm-up (pre-cooling) and at the half-time (per-cooling). The trials will follow a randomized counterbalanced crossover design, single blinded, and will take place in indoor facilities, with Wet Bulb Globe Temperature (WBGT) > 30 °C, at the same time of the day to control for circadian variations.

**Impact of the project:**

The results of this study are expected to determine whether mouth rinsing a menthol solution before and during a football exercise protocol performed in the heat will alter perceptual measures and help ease physiological strain and attenuate performance decrements in elite male football referees, comparing to a non-cooling strategy. Thus, we can be closer to defining nutritional strategies of internal cooling that may be an advantage for the performance of the football referees in the heat.

**Trial registration:**

www.ClinicalTrials.gov NCT05632692 registered on 20 November 2022.

## 1 Introduction

Sports competitions take place in a great diversity of geographical areas, some characterized by hot environments, being anticipated that an increasing number of competitions are and will be held in adverse ambient conditions due to climate change, which is expected to aggravate over the coming years [1]. For instance in Europe, football matches have been reported to be played in extreme environmental temperatures ranging from −5 °C in Norway, to +30 °C in Spain [2]. Moreover, to facilitate the congested European league season, the FIFA World Cup and the UEFA European Championship are usually played during the summer, when temperatures can exceed 35 °C, with potentially high levels of humidity [3,4].

Thermal challenges in sports events carried out under heat stress might influence athletic and cognitive performance, through an increase in core temperature (Tcore) and individual sweating rate (SR) responses, especially for those who live and perform in more temperate climates [5]. As a consequence of the rise of the SR, the risk of dehydration is significantly increased [6]. Dehydration is detrimental to thermoregulatory function during exercise in the heat, resulting in an inhibition of reflex heat dissipation mechanisms of sweating and cutaneous vasodilation, hampering core body temperature maintenance [7,8].

Referees' physical activity during a football match induce a mean total body water loss of 1.60 ± 0.13 L (i.e, mild 2.0% dehydration) [9]. Such a level of dehydration, induced by water restriction, heat, or physical exertion (or a combination of these), cause a reduction in physical [10], psychomotor [11], and cognitive performances, even in temperate environments (22.0 °C ± 2.0 °C, 60.0%–70.0% relative humidity) [12]. In moderately high environmental temperatures (∼30 °C), sprint and jump performances decreased markedly in the last 15 min of a football match [[13], [14], [15]]. 10.13039/100014337Furthermore, a marked reduction in high-intensity running has been observed in a standardised set-up in matches played in hot environments [15], and supported by retrospective analyses of the 2014 FIFA World Cup [16].

During football competition in extreme temperatures, players can modify their game in order to maintain performance and prevent fatigue [17], through the flexibility to adjust game intensity, especially when they are “off-the-ball.” Referees, though, have to follow the rhythm of the match and maintain closeness to match events so that correct decisions can be made [18]. Therefore, although previous research showed that the physiological and physical demands of elite-standard football referees are similar to those of a midfield football player [19,20], these demands may increase under more extreme temperatures since they cannot use the same coping strategies [3]. Thus, heat stress may have detrimental effects on performance and sudden or extreme exposure is a major health concern in this population [15,16].

Several thermal (cooling) strategies have been tested and used with the primary goal of reducing central temperature and thermal sensation (TS), and further delaying the onset of hyperthermia-induced fatigue [21]. These can be applied externally (e.g., using a cooling vest, ice pack, cold-water immersion, topically applied menthol) or internally (e.g., ice slurry, crushed ice, cold beverage ingestion, menthol mouth rinsing) [22]. As a matter of fact, ice slurry ingestion has shown to decrease central and brain temperature, and to improve thermal perception via the stimulation of thermoreceptors located within oral and abdominal regions [22,23]. This has led to an increase in the use of this method in professional competitions performed in hot and humid environments [24].

Research on non-thermal internal cooling methods has increased recently, with particular attention to L-menthol, due to its properties in relieving thermal strain associated with exercise in the heat. Menthol oral application stimulates the mandibular and maxillary branches of the trigeminal nerve (which are predominantly responsible for the detection of temperature and nociceptive stimuli across the face and within the oral cavity) and has consistently shown to improve thermal comfort (TC) and decrease TS, which are thought to modulate perceived exertion to improve performance in the heat [24,25].

A recent systematic review found that rinsing a menthol solution (0.01%) during exercise in the heat significantly improved physical performance (by 3.6–34.4%), mainly in continuous exercises. Conversely, less favourable results were reported for thermal techniques, with less than half of the studies revealing significant improvements in physical performance [26]. However, most of the clinical trials carried out in this area predominantly involve endurance exercise protocols with recreational athletes. It has been pointed out that it is urgent to evaluate the effect of internal cooling strategies on intermittent exercises, especially in elite sports. In this context, the studies carried out concerning internal cooling strategies did not include referees, despite a negative impact of heat stress on both physical and cognitive performance being already well established.

Therefore, the present manuscript intends to propose a protocol for a randomized crossover trial, which aims to analyse the effects of a menthol solution mouth rinse, comparing to a non-cooling strategy, on perceptual, physical and physiological performance responses, in elite male football referees, during a standardised laboratory exercise protocol performed in the heat that aims to mimic the intermittent and multi-directional nature of running in football match-play.

## 2 Methods and analysis

The study has been approved by the Ethics Committee of the Faculty of Nutrition and Food Sciences of University of Porto, Portugal (Report 112/22 CEFCNAUP 2022) and will be conducted in accordance to the declaration of Helsinki for human studies [27]. The study protocol was developed considering the guidelines from Consolidated Standards of Reporting Trials (CONSORT) [28] and the Recommendations for Interventional Trials (SPIRIT) [29].

### 2.1 Sample size and recruitment

For sample size and statistical power calculations, power analysis was based on changes in TS after the internal administration via mouth-rinsing or ingestion of menthol solution. Considering a type I error of 5% and a power of 90%, with a statistical significance (p-value ≤0.05) and a moderate effect size of 0.54 (differences in TS were considered following Jeffries and Waldron results [30]), a total of 10 participants will be needed (using G*Power 3.1.9.2 ®). Considering a drop-out rate of 30% throughout the study, 13 participants as a buffer against attrition.

The Portuguese Football Federation (PFF) will contribute by recruiting referees through direct contact, databases, and referral sources. Following an initial telephone screen to determine the eligibility of potential participants, an orientation session (introductory meeting) will be scheduled to offer detailed information about the study, including the number and type of assessments, the length and nature of the exercise training, and the time commitment required to complete the study. After eligibility criteria confirmation, all participants will sign an informed consent form before being allocated to the study. Participants’ privacy and confidentiality will be guaranteed during and after investigation according to the Portuguese data protection law. Privacy, anonymity, and confidentiality of data/information identifying the participants will be strictly maintained. All information and results of the tests performed on the participants will be confidential. No one other than the investigators of this research will have access to the data.

### 2.2 Participants characteristics and eligibility criteria

Participants meeting the following inclusion criteria will be recruited.

1. Highly trained male field football referees registered in the Portuguese Football Federation aged ≥18 and ≤45 years;
2. With normal weight (Body Mass Index ≥18.5 and ≤24.9 kg/m^2^);
3. Availability to participate in the introductory meeting, familiarization session and 2 experimental sessions;
4. Ability to read and agree to sign the informed consent.

Participants with at least one of the following criteria will be excluded from the study.

1. Under the influence of any medications that may affect urinary parameters, thermoregulation mechanisms, circulatory system, thyroid and pituitary function or metabolic status;
2. Injury, diabetes, autoimmune disease, cardiovascular disease or obstructive disease of the gastrointestinal tract (e.g., diverticulitis, inflammatory bowel disease);
3. Schizophrenia, bipolar disorder or other psychotic disorders;
4. Diagnosed eating disorders;
5. Magnetic resonance imaging (MRI) scans performed within 48 h after familiarization session or any of the experimental trials.

### 2.3 Study design, setting and randomization

A randomized counterbalanced crossover trial with two conditions will be carried out. After passing the eligibility criteria and attending the introductory meeting and a familiarization visit, the participants will be assigned to 2 experimental days for undergoing 2 different randomly ordered experimental conditions. Each condition will be comprised of a 90-min football protocol (SAFT 90) [31], with the administration of one of 2 beverages before warm-up (5 min of pre-cooling) and at the half-time (5 min of per-cooling). There will be a minimum washout period of 7 days between each trial, to reduce carryover effects from the previous condition and to assure an adequate exercise recovery. The trials will take place in an indoor environment, with Wet Bulb Globe Temperature (WBGT) values exceeding 30 °C, at the same time of the day, to control for circadian variations.

After the familiarization visit, the allocation sequence for the order of the experimental conditions will be assigned through individual randomization from a computer-generated random order, equally assigned to every participant, by the main researcher. Randomization will be concealed through a password-protected document that will not be revealed until the first trial. The main researcher will not be blinded in this type of study condition and will have access to the allocation sequence list and will retrieve the randomization code prior to each experimental to prepare the beverages. All the other investigators and the outcomes assessors will be blinded to study condition and group allocation, as well as participants will be blind to the beverages content and aim of the study.

### 2.4 Procedures

#### 2.4.1 Beverage preparation

Beverage A (placebo) is going to be prepared using a noncaloric berry-flavored sweetener consisting of sucralose.

Beverage B (menthol solution) is going to be formulated by crushing and dissolving noncalorific menthol lozenge in warm deionized water, to obtain a solution with a concentration of 0.01%.

Prior to use, both solutions are going to be aliquoted for mouth rinse and warmed at room temperature. They will be served to each participant in individual bottles (75 ml at pre-cooling and 75 ml at per-cooling) and participants will be told to mouth rinse the beverage for 10 s and then to spit-out.

#### 2.4.2 Introductory meeting

A previous presentation is going to be carried out by the research team to explain the study in detail to the participants. All stages of the study will be described, as well as the scales and procedures that are going to be adopted. Participants will be informed that they will have to swallow a telemetric pill 60 min before each trial, and to avoid consumption of alcohol and caffeinated products for 24 h before each trial, as well as strenuous exercise 48 h before testing. The participants will also be asked to drink 2–3 L of water in the day before each session. After understanding and agreeing with the information provided, the participants will sign the informed consent.

#### 2.4.3 Familiarization session

All participants will undergo a familiarization session. All experimental procedures will again be fully described and tested in the field, so that the familiarization trial is as close as possible to the experimental trials resembles. The procedures and timings will be explained to participants.

#### 2.4.4 Exercise protocol

The SAFT90 protocol emulates the intermittent and multi-directional nature of football match-play, with frequent changes in direction and activity [32]. It is based on time-motion analysis data from the English Championship level match play acquired during the 2007 season [31], and simulates the activity demands and physiological responses of football match play [33]. Players navigate around a 20 m agility course in an intermittent fashion via standing (0 km h^−1^), walking (5.5 km h^−1^), jogging (10.7 km h^−1^), striding (15.0 km h^−1^) or sprinting (maximal effort). The players cover 11.1 km in total, 18.5% of this distance (2.04 km) is performed at high-speed (≥15 km h^−1^) with 1269 changes in speed (every 4.3 s), and 888 changes in direction (180°) and 444 cutting manoeuvres over the 90 min (1332 directional changes). The protocol is divided into equivalent 15-min activity profiles, lasts 90 min and is performed on an indoor running surface. The type of movement activity and intensity is controlled using verbal signals from an audio MP3 file [31].

The participants of the present study will perform a standardized pre-match routine in terms of rest, nutrition, hydration and physical preparation and a 15-min warm-up will precede the 90-min simulation, which is going to be interceded by a 15-min passive half-time interval.

#### 2.4.5 Environmental conditions measurement

The trial will be performed in an indoor facility, under high environmental stress conditions asper FIFA's guidelines: a Wet Bulb Globe Temperature (WBGT) between 29.4 and 32.1 °C [34]. Temperature, Relative Humidity and WBGT are going to be measured recurring to Kestrel 5400 Heat Stress Tracker (Kestrel Instruments®, Boothwyn, Pennsylvania, USA). In order to understand a possible heat acclimatization interference in the trial results, Temperature, Relative Humidity and WBGT are going to be recorded in the two days prior to the tests.

#### 2.4.6 Outcomes

The primary outcomes of interest for this study involve physiological, physical and perceptual parameters. Physiological primary outcomes include heart rate (HR), SR and Tcore.

##### 2.4.6.1 Heart rate

HR will be measured through HR monitors (Polar H10 Heart Rate Sensor, USA). PolarH10 can accurately measure mean HR and low-frequency oscillations (up to 0.15 Hz) of HR at rest and during the protocol [35].

##### 2.4.6.2 Sweating rate

SR is going to be calculated recurring to the following equation [36]:

$$SR=\frac{Pre\;exercise\;body\;weight-postexercise\;body\;weight+fluid\;intake-urine\;volume}{exercise\;time\;in\;hours}$$

Pre-exercise body weight will be measured after participants voiding their bladder. Body weight will be measured on a digital platform scale (Seca, Hamburg, Germany), with minimal clothing, to the nearest 0.1 kg. Post-exercise body weight is going to be evaluated also with minimal clothing, after participants' towel-off themselves, at the half-time, before and after urine excretion (if needed), and at the end of the protocol. Fluid intake is going to be recorded via the measurement of mass change of the individual bottles, provided to the participants, at the warm-up and at the end of the first “water break” at the end of the session, to the nearest 0.1 ml (Seca, Hamburg, Germany). Urine volume will be determined through the difference of the referees' weight before and after urine excretion, using a digital platform scale. Participants will be advised not to rinse and spit out the fluids from the bottle at any time of the trial and that they should not drink any other beverages other than those provided by the researchers. Also, fluid intake will only be allowed in the 30th minute of each half, to simulate “cooling break” (“water break”) rule implemented by FIFA. FIFA's guidelines for extreme heat conditions (i.e. a WGBT of more than 32 °C) refer cooling breaks are mandatory in both halves of a match, around the 30th minute and 75th minute, so that football players and referees may rehydrate [37].

##### 2.4.6.3 Core temperature

Tcore will be evaluated through the entire trial through a telemetric pill ingested 60 min prior the start of the session (BodyCap®, Hérouville-Saint-Clair, France). Due to the good measuring accuracy, the ability to measure in field-based situations and the non-invasive character of this temperature measurement method, the ingestible telemetric temperature pill is suitable to assess Tcore during exercise [38].

##### 2.4.6.4 Physical performance

Physical performance responses will be measured using 100 Hz accelerometery, with Sonda 4.0 software (StatSports®, London, UK) to estimate total distance, sprint distance, accelerations, and decelerations.

##### 2.4.6.5 Perceptual parameters

Perceptual responses that are going to be evaluated in this study include 4 subjective scales: RPE, TC, perceived thirst (PT) and TS. RPE will be recorded through CR-10 Borg scale [39] that goes from 0 (“rest”) to 10 (“maximal effort”). TC is going to be assessed according to a 6-point scale from to −3 (“very uncomfortable) to 3 (“very comfortable”) [40]. PT will be evaluated recurring to a 7-point scale from 1 (“not thirsty at all”) to 7 (“very, very thirsty”) [27]. TS will be recorded with a 9-point scale (33) from −4 (“very cold”) to 4 (“very warm”) [41].

Secondary outcomes of this study include hydration status, *ad libitum* fluid intake during “water breaks”, lactate, and glucose blood levels.

##### 2.4.6.6 Hydration status

Hydration status is going to be assessed through urine specific gravity, recurring to urine test strips (Combur10 Test M, Roche, Switzerland) and a Urisys 1100® analyzer (Roche, Switzerland) before the start and at the end of the session. Hydration status will also be evaluated through urine colour assessment, by a trained researcher, according to Armstrong scale [36].

##### 2.4.6.7 Ad libitum fluid intake

*Ad libitum* fluid intake allowed on “water break” time is going to be recorded through the same procedures used to estimate fluid intake as previously described methods to assess SR evaluation.

##### 2.4.6.8 Blood lactate and glucose levels

Blood lactate and glucose levels will be measured before and after the protocol, recurring to a Blood Lactate Meter (Lactate Pro 2, Arkray, U.S.A) and a Glucometer (FreeStyle Precision Neo, Abbott Laboratories, U.S.A), respectively. Both devices have been previous validated [42,43].

#### 2.4.7 Experimental trial protocol

**Fig. 1:**
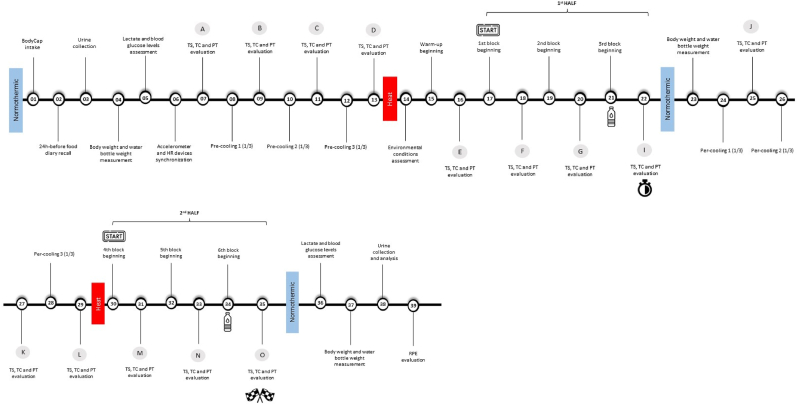
Schematic representation of the experimental protocol.

**Fig. 2:**
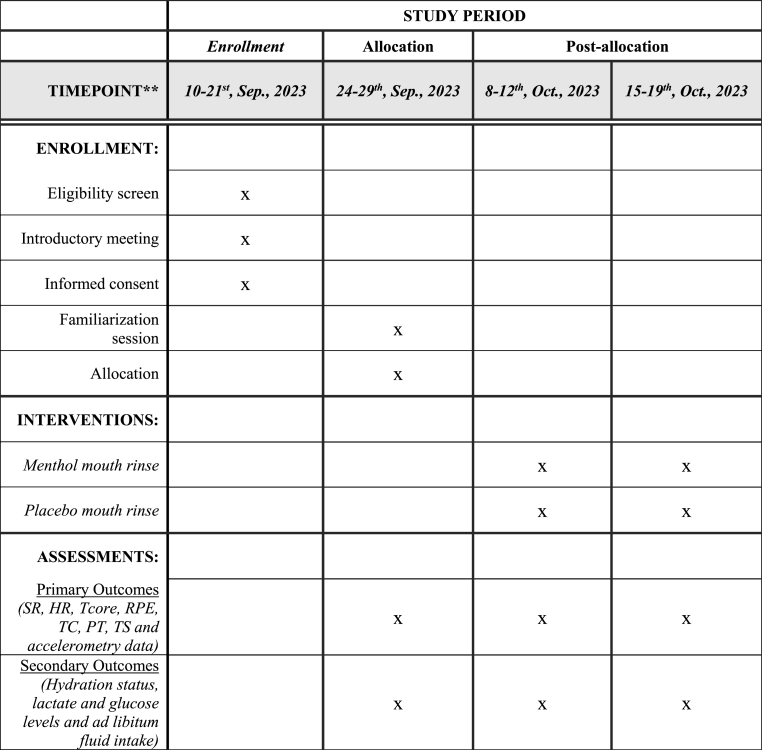
Schedule of enrolment, interventions, and assessments, according to the SPIRIT guidelines.

The protocol of the trial will be divided into 39 phases (T1-T39) (Table 1 and Fig. 1). The participants will arrive at the testing facilities 1 h before the start of the warm-up. Schedule of the enrolment, interventions, and assessments is described in Fig. 2.

**Table 1:** Description of the experimental trial phases.

**T1**	BodyCap® will be ingested by the participant.
**T2**	Then, participant's 24-h food recall diary will be collected.
**T3**	Hydration status will be assessed, after urine collection.
**T4**	After voiding their bladder, body mass will be measured on a digital platform scale, weighted with minimal clothing. Individual marked drink bottles will be distributed to the referee so that fluid consumed at “water breaks” may be recorded via the measurement of body mass changes. Participants will be informed that they may drink water *ad libitum*, on “water breaks”.
**T5**	Lactate and blood glucose levels are now going to be measured.
**T6**	Devices for assessing HR and physical performance parameters will now be placed on the referee and synchronized.
**T7**	TC, PT and TS are will be measured.
**T8**	Beverages (previously prepared and stored at the intended temperature) will be distributed to the referee. Total amount of beverages for pre-cooling previously described (75 ml of beverage A or B) will be divided into 3 equal parts and made available to the participant every 1 min. Beverages temperature will be monitored with a digital thermometer, to the nearest 0.1 °C (Proficook, Germany). The entire cooling stage will be carried out at a mild temperature of 18–20 °C, at rest. Participants will be instructed to swill both beverages for 10-s before spitting into a bowl without swallowing. The first 1/3 of the 75 ml of beverage will be administered.
**T9**	TC, PT and TS will be again evaluated.
**T10**	The second 1/3 of the 75 ml of pre-cooling beverage will be distributed.
**T11**	TC, PT and TS will be recorded.
**T12**	The third 1/3 of the 75 ml of pre-cooling beverage will be administered.
**T13**	TC, PT and TS will be again evaluated.
**T14**	Ambient temperature, relative humidity, and heat stress and WBGT will be recorded.
**T15**	Participants will start the 15-min warm-up.
**T16**	After the warm-up, TC, PT and TS will be again evaluated.
**T17**	The first 15-min block of the first half will start.
**T18**	TC, PT and TS will be recorded again after the end of the first block.
**T19**	The second 15-min block of the first half will begin.
**T20**	TC, PT and TS will be evaluated after the end of the second block.
**T21**	First “water break” is now going to occur. The third 15-min block of the first half will start.
**T22**	TC, PT and TS will be assessed after the end of the third block and half-time period is going to start.
**T23**	If needed, participants will towel-dry themselves and then have their body mass measured (before and after urine excretion) with the same scale and while wearing the same clothes as the pre-exercise body mass assessment. Water bottles will be weighted.
**T24**	The same beverage (as in T8) will be provided to each participant, exactly in same conditions as in the pre-cooling phase. The first 1/3 of the 75 ml of the same beverage will be administered.
**T25**	TC, PT and TS will be recorded.
**T26**	The second 1/3 of the 75 ml of per-cooling beverage will be distributed.
**T27**	TC, PT and TS will be evaluated.
**T28**	The third 1/3 of the 75 ml of per-cooling beverage will be administered.
**T29**	TC, PT and TS will be accessed.
**T30**	The first 15-min block of the second half will start.
**T31**	TC, PT and TS will be recorded again after the end of the fourth block.
**T32**	The second 15-min block of the second half will begin.
**T33**	TC, PT and TS will be evaluated after the end of the fifth block.
**T34**	Second “water break” will occur. The third 15-min block of the second half will start.
**T35**	TC, PT and TS will be evaluated at the end of the sixth block.
**T36**	Lactate and blood glucose levels are going to be reassessed.
**T37**	Participants will towel-dry themselves and then have their body mass measured (before urine excretion) with the same scale and while wearing the same clothes as the pre-exercise body mass assessment. Water bottles will be weighed. Subsequently, the difference between the referee's weight at T4 and T37 is going to be calculated, to estimate whole-body sweat loss as well as SR during the protocol (the volume of fluid intake is going to be subtracted, as well as urine output).
**T38**	Hydration status is going to be evaluated, after urine collection.
**T39**	RPE will be assessed.

### 2.5 Data management

All participants will be assigned a unique identifier to protect they identify. All personal data will be stored in encrypted files with access restricted to the main researcher. Original paper files will be stored in secure, locked cabinets onsite. All data will be inserted electronically. Data will be double-checked for errors at the time of entry, with all changes performed being documented. Additional findings will be performed through statistical software. Data ranges and consistency checks will be performed for data integrity. No specific auditing procedures are planned for this trial. The results will be fully disseminated in peer-reviewed scientific journals and conferences.

### 2.6 Adherence promotion efforts

To increase adherence and promote the compliance of participants in the familiarization session and in each one of the 2 experimental trials, different strategies will be adopted namely logistical support (compensation for travel reimbursement) and collaboration between subjects and research staff (with regular phone calls and messages with positive reinforcement). Also, in the day before the familiarization session and the 2 experimental trials, the researchers will reach participants by phone, to gently remind them of the importance of participating in the study.

### 2.7 Statistics

Statistical analysis will be performed using IBM SPSS statistics version 27.0, 2020 (SPSS Inc., an IBM Company, Chicago, IL, USA). To test the normality of distribution of the variables, the Shapiro-Wilk test will be performed. Baseline demographic and clinical characteristics of the participants will be analysed using descriptive statistics (mean, standard deviation, and range). For the continuous normal distributed variables, the one-way repeated measures analysis of variance (ANOVA) will be used to assess the association between the intervention and the perceptual, physical and physiological responses (thermal sensation, thermal comfort, perceived thirst, HR, accelerometery data, Tcore, SR, *ad* libitum fluid intake, lactate and glucose blood levels). Single time-point data will be examined for within-group effects across conditions using a one-way ANOVA. A two-way analysis repeated measures ANOVA will be used to test for within-group effects across time in both conditions. Missing data will not be included in the statistical analysis. Participants who discontinue or deviate from intervention protocols, and who meet exclusion criteria at some point of the intervention period, will be excluded. A *p* value of 0.05 will be considered statistically significant. Data will be reported according to the guidelines of consolidated standards of reporting trials (CONSORT) [28].

## 3 Discussion

The present research protocol intends to provide insights on the effect of the administration of oral menthol before and during intermittent exercise in the heat, in elite football referees. Due to the rules, regulations, and characteristics of the football game, to date, the evidence-based cooling options to be implemented in real-world are still limited. Though, in football, cooling strategies should be practical and cost-effective, and internal cooling strategies seem to meet these requirements, possibly assisting players and referees to cope with heat, mitigating the effects of hyperthermia and hypohydration on exercise performance.

Even though the negative consequences of exercise in the heat are an already established problem by the scientific community, internal cooling methods have been mainly evaluated on endurance exercise and mainly with recreational athletes. More research is required on intermittent efforts and in elite athletes, especially regarding non-thermal internal cooling techniques [25].

Based on previous studies, we suggest that the thermal stress and fatigue experienced by football referees in extreme environments may interfere with their TS and TC, resulting in decreased physical performance [3]. Mouth-rinsing a menthol solution, a non-thermal internal cooling technique, may be used as a nutritional cooling intervention prior to and/or during exercise in hot conditions. The improvements in RPE, TC, and TS in response to menthol oral administration may increase the thermal tolerance, which can lead to a better performance [21,25,44]. Also, mouth-swilling strategies may be useful during exercise to alleviate ‘dry mouth’ brought about by a reduction in salivary flow rate [45]. This use is fitting, with menthol shown to increase the drive to breathe [46], elevate ventilation [47] and attenuate thirst [48], along with eliciting sensations of coolness and freshness that may alleviate thermal symptoms during exercise [49]. However, as mentioned before, the ergogenic benefits of menthol have been essentially observed in fixed intensity/perceptual or tolerance-based protocols, and further research is required among other training backgrounds.

Mouth-rinsing a menthol solution throughout exercise has shown to improve time to exhaustion [[50], [51], [52], [53]], power output [50,52,54], completion time in a time trial [54,55] in other studies in laboratory context within a different range of participants. As such, we posit that menthol will also benefit sports performance in an intermittent protocol with elite referees. Additionally, TS [50,52] and comfort rating scales [50], and physiological strain [53,55] are also expected to improve compared to the exercise under the same conditions without any cooling strategy, as other studies suggest.

Oral menthol administration has been more frequently investigated as a per-cooling technique. Most studies assumed that this method will only have an ergogenic effect if applied during more advanced stages of exercise. However, in one study, the authors opted to apply menthol mouth rinsing 1 min before the start of a 3-min aerobic test, and found significant improvements in physical performance [56]. While in internal thermal cooling methods, it is important that pre-cooling is carried out in advance to see improvements in heat storage, in the case of menthol it seems that pre-cooling can be performed very shortly before the start of the protocol [56]. This increases the likelihood of immediately improving the TS and TC in the initial phases of short exercises. So, for those reasons, a pre- and per-cooling mode will be chosen to administrate oral menthol to the referees in the current research protocol. This way, we will be able to understand the effects of menthol even at the earliest stages of exercise.

The main strength of the present study is the design. By using a crossover trial to compare the interventions, we can minimize the risk of confounding because each participant will be their own control. On the other hand, counterbalancing will allow to control the effects of nuisance variables (e.g., small differences on ambient conditions between the trials, acclimatization effect, and beverages selection order bias), thus enhancing the study's interval validity.

However, the protocol of the study has some limitations. First, it will be very difficult to completely blind the participants to the beverage content, due to the sensorial characteristics of the intervention. Participants will not be told the content of the beverages and will be blinded to the aim of the study (i.e., they will be told that the purpose of the study is to evaluate the effect of the heat on physiological parameters, such as Tcore and HR). We are also expecting to find some barriers in retaining the participants through the study. To minimize this risk, participants will be frequently contacted during the wash-out period to motivate and remember about the following trial. Additionally, SAFT-90 protocol is validated for football players, not for referees. However, in order to achieve similar ambient conditions throughout the trials, the authors opted for an indoor protocol, where these conditions can be controlled. And, to the best of our knowledge, there are no laboratory and field-based match play simulations protocols validated for referees. It is also important to consider that this experimental protocol only allows the administration of the beverages before the protocol and at the half-time, leaving the referees for a long time (45 min) without access to any cooling method, besides the 1-min “cooling break” for hydration. However, this study pretends to apply a standardised laboratory exercise protocol designed to mimic the intermittent and multi-directional nature of running in football match-play, as well as game timings. So, also in a real game context, the administration of internal cooling strategies will only be possible before the start of the match-play, in “cooling breaks” (only in high temperature and relative humidity conditions, often 30 min after the start of the first and second halves, and at the half-time. In this case, as the present study sought to mimic the real game context as much as possible, the "cooling breaks" have the purpose to hydrate, so it would not be possible to rinse with menthol solution, or even the occurrence of both at the same time could generate confounding results. Finally, this is the first study of this kind therefore subsequent studies will be required to confirm the results.

**Trial status:** This is the first trial Protocol version and the trial is not yet ongoing. The recruitment is going to start and end in September 2023. We expect the end of the study to take place by October 2023.

## Funding

This research did not receive any specific grant from funding agencies in the public, commercial, or not-for-profit sectors.

## Availability of data and materials

Not applicable.

## Authors’ contributions

Conceptualization, M.R., J.B. and V.H.T.; methodology, M.R., J.B., F.J.T., K.S. and V.H.T.; software, J.B., F.J.T., and K.S.; validation, J.B., F.J.T., K.S. and V.H.T.; writing, M.R.; writing review and editing, J.B., F.J.T., K.S. and V.H.T. All authors have read and agreed to the published version of the manuscript.

## Declaration of competing interest

The authors declare that they have no known competing financial interests or personal relationships that could have appeared to influence the work reported in this paper.

## References

[1] NASA/GISS *Global land-ocean temperature index* may 4, 2021]. https://climate.nasa.gov/.

[2] Taylor L. (2014). Exposure to hot and cold environmental conditions does not affect the decision making ability of soccer referees following an intermittent sprint protocol. Front. Physiol..

[3] Gaoua N., de Oliveira R.F., Hunter S. (2017). Perception, action, and cognition of football referees in extreme temperatures: impact on decision performance. Front. Psychol..

[4] Schneider S. (2022). Physical activity, climate change and health-A conceptual model for planning public health action at the organizational level. Int. J. Environ. Res. Publ. Health.

[5] Donnan K., Williams E.L., Stanger N. (2021). The effects of heat exposure during intermittent exercise on physical and cognitive performance among team sport athletes. Percept. Mot. Skills.

[6] Baker L.B. (2017). Sweating rate and sweat sodium concentration in athletes: a review of methodology and intra/interindividual variability. Sports Med..

[7] Fortney S.M. (1984). Effect of hyperosmolality on control of blood flow and sweating. J. Appl. Physiol. Respir. Environ. Exerc. Physiol..

[8] Sawka M.N. (1998). Hydration effects on temperature regulation. Int. J. Sports Med..

[9] Da Silva A.I., Fernandez R. (2003). Dehydration of football referees during a match. Br. J. Sports Med..

[10] Krustrup P. (2009). Activity profile and physical demands of football referees and assistant referees in international games. J. Sports Sci..

[11] Epstein Y. (1980). Psychomotor deterioration during exposure to heat. Aviat Space Environ. Med..

[12] Irwin C. (2013). The effects of dehydration, moderate alcohol consumption, and rehydration on cognitive functions. Alcohol.

[13] Mohr M. (2010). Examination of fatigue development in elite soccer in a hot environment: a multi-experimental approach. Scand. J. Med. Sci. Sports.

[14] Kurdak S. (2010). Hydration and sweating responses to hot-wather football competition. Scand. J. Med. Sci. Sports.

[15] Mohr M. (2012). Physiological responses and physical performance during football in the heat. PLoS One.

[16] Nassis G.P. (2015).

[17] Racinais S. (2012). Individual responses to short-term heat acclimatisation as predictors of football performance in a hot, dry environment. Br. J. Sports Med..

[18] Catterall C. (1993). Analysis of the work rates and heart rates of association football referees. Br. J. Sports Med..

[19] Casajus J.A., Castagna C. (2007). Aerobic fitness and field test performance in elite Spanish soccer referees of different ages. J. Sci. Med. Sport.

[20] Lohman T.G., Roche A.F., Martorell R. (1988).

[21] Ruddock A. (2017). Practical cooling strategies during continuous exercise in hot environments: a systematic review and meta-analysis. Sports Med..

[22] Bongers C.C. (2015). Precooling and percooling (cooling during exercise) both improve performance in the heat: a meta-analytical review. Br. J. Sports Med..

[23] Siegel R., Laursen P. (2011). Keeping your cool possible mechanisms for enhanced exercise performance in the heat with internal cooling methods. Sports Med..

[24] Racinais S. (2022). Association between thermal responses, medical events, performance, heat acclimation and health status in male and female elite athletes during the 2019 Doha World Athletics Championships. Br. J. Sports Med..

[25] Barwood M.J. (2020). Menthol as an ergogenic aid for the Tokyo 2021 olympic games: an expert-led consensus statement using the modified delphi method. Sports Med..

[26] Roriz M. (2022).

[27] (2013). World Medical Association Declaration of Helsinki: ethical principles for medical research involving human subjects. JAMA.

[28] Dwan K. (2019). CONSORT 2010 statement: extension to randomised crossover trials. BMJ.

[29] Chan A.W. (2013). SPIRIT 2013 statement: defining standard protocol items for clinical trials. Ann. Intern. Med..

[30] Jeffries O., Waldron M. (2019). The effects of menthol on exercise performance and thermal sensation: a meta-analysis. J. Sci. Med. Sport.

[31] Small K. (2010). The effects of multidirectional soccer-specific fatigue on markers of hamstring injury risk. J. Sci. Med. Sport.

[32] Barrett S., Guard A., Lovell R. (2013).

[33] Lovell R. (2013). Effects of different half-time strategies on second half soccer-specific speed, power and dynamic strength. Scand. J. Med. Sci. Sports.

[34] Nassis G. (2015). The association of environmental heat stress with performance: analysis of the 2014 FIFA World Cup Brazil. Br. J. Sports Med..

[35] Hernández-Vicente A. (2021). Validity of the polar H7 heart rate sensor for heart rate variability analysis during exercise in different age, body composition and fitness level groups. Sensors.

[36] Armstrong L.E. (2007). Assessing hydration status: the elusive gold standard. J. Am. Coll. Nutr..

[37] FIFA. *Football development*, players' health Playing in the heat. http://www.fifa.com/development/medical/players-health/minimising-risks/heat.html.

[38] Bongers C.C., Hopman M.T., Eijsvogels T.M. (2015). Using an ingestible telemetric temperature pill to assess gastrointestinal temperature during exercise. J. Vis. Exp..

[39] Borg G. (1970). Perceived exertion as an indicator of somatic stress. Scand. J. Rehabil. Med..

[40] Bedford T. (1936).

[41] Zhang H. (2004). Thermal sensation and comfort in transient non-uniform thermal environments. Eur. J. Appl. Physiol..

[42] Raa A. (2020). Validation of a point-of-care capillary lactate measuring device (Lactate Pro 2). Scand. J. Trauma Resuscitation Emerg. Med..

[43] Brannan C. (2015). Evaluation of the FreeStyle precision Pro blood glucose and β-ketone monitoring system. Point Care: The Journal of Near-Patient Testing & Technology.

[44] Kissling L.S., Akerman A.P., Cotter J.D. (2019). Heat-induced hypervolemia: does the mode of acclimation matter and what are the implications for performance at Tokyo 2020?. Temperature (Austin).

[45] Dawes C. (1987). Physiological factors affecting salivary flow rate, oral sugar clearance, and the sensation of dry mouth in man. J. Dent. Res..

[46] Eccles R. (2003). Menthol: effects on nasal sensation of airflow and the drive to breathe. Curr. Allergy Asthma Rep..

[47] Meamarbashi A., Rajabi A. (2013). The effects of peppermint on exercise performance. J Int Soc Sports Nutr.

[48] Eccles R. (2000). Role of cold receptors and menthol in thirst, the drive to breathe and arousal. Appetite.

[49] Stevens C.J., Best R. (2017). Menthol: a fresh ergogenic aid for athletic performance. Sports Med..

[50] Flood T.R., Waldron M., Jeffries O. (2017). Oral L-menthol reduces thermal sensation, increases work-rate and extends time to exhaustion, in the heat at a fixed rating of perceived exertion. Eur. J. Appl. Physiol..

[51] Mündel T., Jones D.A. (2010). The effects of swilling an L(-)-menthol solution during exercise in the heat. Eur. J. Appl. Physiol..

[52] Parton A.J. (2021). Thermo-behavioural responses to orally applied l-menthol exhibit sex-specific differences during exercise in a hot environment. Physiol. Behav..

[53] Jeffries O., Goldsmith M., Waldron M. (2018). L-Menthol mouth rinse or ice slurry ingestion during the latter stages of exercise in the heat provide a novel stimulus to enhance performance despite elevation in mean body temperature. Eur. J. Appl. Physiol..

[54] Gavel E.H. (2021). Menthol mouth rinsing and cycling performance in females under heat stress. Int. J. Sports Physiol. Perform..

[55] Riera F. (2014). Physical and perceptual cooling with beverages to increase cycle performance in a tropical climate. PLoS One.

[56] Crosby S. (2022). Menthol mouth rinsing maintains relative power production during three-minute maximal cycling performance in the heat compared to cold water and placebo rinsing. Int. J. Environ. Res. Publ. Health.

